# Pyrethroids Differentially Alter Voltage-Gated Sodium Channels from the Honeybee Central Olfactory Neurons

**DOI:** 10.1371/journal.pone.0112194

**Published:** 2014-11-12

**Authors:** Aklesso Kadala, Mercedes Charreton, Ingrid Jakob, Thierry Cens, Matthieu Rousset, Mohamed Chahine, Yves Le Conte, Pierre Charnet, Claude Collet

**Affiliations:** 1 INRA, UR 406 Abeilles et Environnement, Toxicologie Environnementale, Avignon, France; 2 CNRS, UMR 5237, Centre de Recherche de Biochimie Macromoléculaire, Université Montpellier 2, Montpellier, France; 3 Department of medicine, Laval University, Québec city, Canada; 4 UMT Protection des Abeilles dans l'Environnement, Avignon, France; University of Missouri, United States of America

## Abstract

The sensitivity of neurons from the honey bee olfactory system to pyrethroid insecticides was studied using the patch-clamp technique on central ‘antennal lobe neurons’ (ALNs) in cell culture. In these neurons, the voltage-dependent sodium currents are characterized by negative potential for activation, fast kinetics of activation and inactivation, and the presence of cumulative inactivation during train of depolarizations. Perfusion of pyrethroids on these ALN neurons submitted to repetitive stimulations induced (1) an acceleration of cumulative inactivation, and (2) a marked slowing of the tail current recorded upon repolarization. Cypermethrin and permethrin accelerated cumulative inactivation of the sodium current peak in a similar manner and tetramethrin was even more effective. The slow-down of channel deactivation was markedly dependent on the type of pyrethroid. With cypermethrin, a progressive increase of the tail current amplitude along with successive stimulations reveals a traditionally described use-dependent recruitment of modified sodium channels. However, an unexpected decrease in this tail current was revealed with tetramethrin. If one considers the calculated percentage of modified channels as an index of pyrethroids effects, ALNs are significantly more susceptible to tetramethrin than to permethrin or cypermethrin for a single depolarization, but this difference attenuates with repetitive activity. Further comparison with peripheral neurons from antennae suggest that these modifications are neuron type specific. Modeling the sodium channel as a multi-state channel with fast and slow inactivation allows to underline the effects of pyrethroids on a set of rate constants connecting open and inactivated conformations, and give some insights to their specificity. Altogether, our results revealed a differential sensitivity of central olfactory neurons to pyrethroids that emphasize the ability for these compounds to impair detection and processing of information at several levels of the bees olfactory pathway.

## Introduction

In social bees, olfaction is a key function that underlies many activities such as nursing, defense against parasites and predators, foraging, and orientation. Antennae play a key role in olfaction as they house olfactory receptor neurons (ORNs) which are responsible for odors and pheromones detection [1], [2]. The second stage of the olfactory pathway involves antennal lobe neurons (ALNs) which are responsible for the processing of olfactory information [3]. Residues of many pyrethroid insecticides (that are commonly used to protect fields from insects considered as pests from an agricultural point of view) have been detected in a number of hives and their outer environment [4], [5]. In insects, the symptoms generally associated with type I pyrethroid (e.g., tetramethrin) poisoning are the absence of coordination, hyperactivity and prostration. In addition, for type II pyrethroids (e.g., cypermethrin), periods of convulsions followed by paralysis are also noticed [6]. It should be noted that the earlier classification as type I or type II initially relied on extreme symptoms of poisoning and thereafter the absence or presence of an alpha-cyano residue, but this structural dichotomy turned out to be simplistic since some compounds show intermediate properties [7]. From a toxicological point of view, the study of sublethal effects of pyrethroids is now a priority since subtle modifications can strongly affect highly complex organizations such as those exhibited by social bees. In the honeybee, these sublethal effects include impairment of olfaction and learning performances [8], [9], behavioral changes such as disorientation and desertion from the hive [10], [11] that would globally lead to colony disturbance. Whereas some of these sublethal effects (especially the so called ‘knockdown’ effect) have been ascribed to their deleterious action on the peripheral nervous system, little direct evidence has been so far obtained in honeybees [12]. Considering their peripheral location, antennal ORNs are likely to be primarily exposed to these neurotoxic insecticides during various activities, especially foraging. In accordance with the peripheral hypothesis, pyrethroids change the action potential activity in ORNs of moths, as demonstrated in electroantennogram and single sensilla recording assays [13]. Some sublethal effects of pyrethroids (e.g., decrease of queen's egg-laying) have also been attributed to their action on the central nervous system [14], and in particular on neurons from the olfactory pathway, such as ALN neurons.

In neurons, pyrethroids primarily target the voltage-gated sodium channels responsible for action potential generation [15], [16]. Most of these studies however rely on voltage-clamp experiments on vertebrate voltage-sensitive channels either in their native environment [17], [18] or heterologously-expressed in *Xenopus* oocytes [19], [20]. To our knowledge, the direct analysis of the effects of pyrethroids on insects sodium channels in their native neuronal environment are very rare under voltage-clamp conditions (for example *Heliothis virescens* [21]) and do not take into account use-dependent processes that are typical of this insecticide class. A large part of the data on insect channels susceptibility came from expression studies (*Xenopus oocytes*) on sodium channels from insects that are considered as pests, such as *Blatella germanica*, *Musca domestica* or *Drosophila melanogaster* [22], [23], [24]. Unfortunately, in the case of honey bee, no cloning and no heterologous expression of the voltage-gated sodium channels have been reported yet. Our group has already studied the effects of pyrethroids on peripheral ORN neurons from the honeybee antenna [12]. The present paper reports on the differential modifications of voltage-gated sodium channels functions by 3 types of pyrethroids in central neurons from identified honeybee brain regions involved in the processing of olfactory information, the antennal lobes, with an emphasis on use-dependent channels modifications.

## Materials and Methods

### Cell culture

Antennal lobe neurons (ALNs) were isolated from the brains of domestic honeybee *A. mellifera* pupae (at stages between four and six days before emergence). Pupae were first dipped in alcohol for few seconds and then rinsed in sterile distilled water for sterility purposes. The brain was dissected out of the insect's forehead in a sterile Ca^2+^ and Mg^2+^ -free Tyrode (400 mOsm/l, see Solutions). The brain sheath was then removed and the antennal lobes were isolated. After a hyperosmotic non-enzymatic dissociation in Ca^2+^ - Mg^2+^ free Tyrode, (500 mOsm/l, 4°C; 15 min) and centrifugation (0.3 g, 3 min), the pellet was suspended (one antennal lobe per 15 µl) in culture medium (see Solutions). Fragments of the antennal lobes were gently triturated through the disposable tip of a p100 pipette. Isolated neurons were plated on poly-L-lysine coated plastic Petri dishes, and cultured within a liquid space formed by a coverslip supported by two pieces of glass spacers attached with a non-cytotoxic silicone grease to the bottom of the Petri dish. Dishes were thus kept upside down in an incubator (29°C, high humidity). All experiments were done on 2 to 5 days-old cell cultures and performed at room temperature (20–22°C).

### Electrophysiology

Membrane currents were measured in the whole-cell configuration using a patch-clamp amplifier (RK400, Bio-Logic, Claix, France). Voltage pulse generation and data acquisition were done using WinWCP software (John Dempster, Strathclyde University, UK) driving an A/D, D/A converter (PCI-6014 board, National Instruments Corp. Austin, TX, USA). Patch-clamp pipettes were pulled from borosilicate glass capillaries on a vertical pipette puller (P30, Sutter Instruments Co, Novato, AS, USA) and then sylgard-coated to minimize pipette capacitance. The resistance of the recording electrode filled with intracellular solution (see Solutions below) ranged between 3 and 5 MΩ in standard extracellular solution. The liquid junction potential (<3 mV) was not corrected. Microelectrode offset potential was nulled before seal formation. After gigaseal formation and before membrane patch rupture, the resting holding potential was established at −80 mV and the residual microelectrode capacitance was nulled with a fast analog compensation circuit available on the amplifier. In the whole cell configuration, capacitance and series resistance were maximally compensated for. Passive leak currents and residual linear capacitative currents were subtracted using a P/4 protocol. Current-voltage relationships were generated by step depolarizations to test potentials from −80 to +70 mV (10 mV increment) from −80 mV. Individual curves of the sodium current voltage dependence were fitted with equation 1: I(V)  =  G_max_(V-V_rev_)/(1+exp[V_0.5_-V)/k]), where I(V) is the peak density of the current for a depolarization to a membrane potential V, G_max_ is the maximum conductance, V_rev_ is the apparent reversal potential, V_0.5_ is the half activation voltage and k is a steepness factor. To explore steady-state inactivation, the membrane potential was stepped from à −80 mV holding potential to a series of 100 ms conditioning prepulses in the range of −100 to +20 mV, followed by a 0 mV test potential. Experimental data were fitted with equation 2: h  = 1/(1 + exp[(V-V_h_)/K_h_]), where V_h_ is the potential for half inactivation and K_h_ a slope parameter.

To assess pyrethroid effects, a single stimulation protocol consisted of a 3 ms depolarization from a holding potential of −80 mV to −10 mV. Trains of repetitive depolarizations at 13 Hz were also used and consisted of ten short stimulations (3 ms to −10 mV) with an interpulse duration (time lapse between the initiation of two successive pulses) of 78 ms. Three milliseconds pulses were selected since short pulses durations allow for the development of the maximal tail currents as demonstrated in other neuronal preparations [25], [26]. The percentage of channels modified by pyrethroids was calculated using Equation 3: M  =  (I_tail_/(E_h_-E_rev_))/(I_Na_/(E_t_-E_rev_)) x 100 where I_tail_ is the maximal tail current amplitude, E_h_ is the potential to which the membrane is repolarized, E_rev_ is the reversal potential for the sodium current, I_Na_ is the amplitude of the current during depolarization (measured in control conditions) and E_t_ is the membrane potential reached during the test pulse [25]. Patch-clamp data were analyzed with OriginPro software. Values are given as mean ± S.E.M. The Student's *t*-test was used to compare means, with a significance level set at *P*<0.05.

### Solutions

The Ca^2+^ and Mg^2+^ -free Tyrode used for dissection contained (in mM): 140 NaCl, 5 KCl, 10 HEPES, 90 sucrose (pH 7.2, adjusted with NaOH, 400 mOsm/l). The hyperosmotic Tyrode contained 190 instead of 90 mM sucrose. Culture medium was made of a commercial liquid L15 medium (with L-glutamine) supplemented with 5.5 mM D-Glucose, 3.3 mM L-proline, 75 mM sucrose, 10% fetal bovine serum, 1% penicillin/streptomycin (pH 7.2, adjusted with HCl, 400 mOsm/l). The standard extracellular solution used for patch-clamp in order to isolate the sodium current contained (in mM): 120 NaCl, 20 TEA-Cl, 2 MgCl_2_, 2 BaCl_2_, 0.1 CdCl_2_, 1 4-aminopyridine, 10 HEPES, 90 sucrose (pH 7.2 ajusted with HCl, 400 mOsm/l). A concentration of 0.1% DMSO was also added to the extracellular solution during the experiments with pyrethroids, to match the concentration present in the insecticide-containing solution. Intracellular (pipette) solution contained (in mM): 135 CsCl, 5 NaCl, 1 MgCl_2_, 1 CaCl_2_, 10 EGTA, 10 HEPES, 90 sucrose (pH 7.2, adjusted with CsOH, 380 mOsm/l). Pyrethroid compounds were purchased from Sigma-Aldrich Co (St-Louis, MO, USA). Stock solutions (10 mM) were prepared in DMSO (thoroughly vortexed) and dissolved in the perfusion solution (concentration in DMSO never exceeded 0.1%, a concentration that did not have any detectable effect on the sodium current). We used a single use glass capillary device as described by Tatebayashi and Narahashi [25] to expose olfactory neurons to pyrethroids. Only one neuron was recorded per dish.

### Modeling

The state model of the sodium channels was constructed using a formalism derived from previous model as well as from available structural and functional data (see Results and Discussion). Four Closed and one Open states represent the gating process that opens the channel. The Closed states refer to the successive voltage-dependent activation (transition rates α and β) of the 3 similar S4 voltage-sensors necessary for channel opening. Transition to the Open state also requires an additional voltage dependent mechanism (rates KFwr, and Kbck, see Results and Discussion). Fast inactivation (hinged-lid -IFM inactivation due to the loop connecting domains III and IV) is also voltage-dependent since it relies on the activation of the 4th voltage sensor (DIVS4, transition rates KiF, KiB, [27]). The deep, slow, pore-dependent inactivation is not dependent on voltage and therefore transition rates are set as constants (Kin and Kout). Voltage-dependent transition rates are set as A.exp^(V/k)^, with A the value at V = 0, k the voltage dependency, and V the membrane potential. The set of differential equations describing the system is solved numerically to fit the experimental data recorded in control conditions, giving a set of values able to reproduce most of the channel properties (see Figure S1). For the effects of pyrethroids, drug binding has been limited to the Open state, as suggested from their use-dependent effects described in a number of articles, and the location of the pyrethroid binding site within the channel pore [28], [29]. We are aware of the possible binding to Closed or intermediate states during the gating process [30] through lateral fenestrations of the channel pore within the plasma membrane for example [31]. In our conditions, this possibility was also explored (see Supplementary Informations), but not presented in the results section because (i) the use-dependent inhibition and the location of the binding site suggest Open state binding, as stated above, (ii) data with tetramethrin that could suggest Closed state binding are better fitted with a model that does not required it, (iii) this pyrethroid is also the most hydrophylic one, which clearly does not favor Closed-state binding on a buried binding site and (iv) using Closed state binding gave qualitatively the same results on the kinetics modifications that affect transitions to Open and Inactivated states (see Supplementary Informations), but changed the Kd values to less sensitive values from tens of nanomoles to micromoles. However, binding to the Open state does not mean therefore that the drugs accesses its binding sites by the hydrophilic pathway of the open pore, but just that the open conformation is required, which, in this case is functionally equivalent. Once bound, the kinetics parameters that can be affected are those that depend on the pore module where the binding site is located (DIS4-S5 linker, DIS5, DIIS6, DIIS4-S5linker, DIIS5, and DIIIS6 [28], [29]) i.e. transition rates to and from Open and Inactivated states. We have limited the possible changes to the amplitude only, leaving voltage-dependency unaffected. The p, q, r, s, t and u parameters are therefore the factors affecting KFwr, KBck, KiF, KiB, Kin and Kout, respectively, giving the drug-bound values KFwrb, KBckb, KiFb, KiBb, Kinb and Koutb. This model was used to fit the experimental data in the presence of cypermethrin, permethrin and tetramethrin, and values of p, q, r, s, t and u were then displayed on a radargraph with a log scale for visualization (see Results and Discussion). The binding and unbinding of the drugs (KPyrF, and KPyrB) were also evaluated and used to calculate the Kd values for each drug. Simulation and fitting were done using the Berkeley-Madonna software.

## Results

### Electrophysiological properties of the sodium current in ALNs

**Figure 1:**
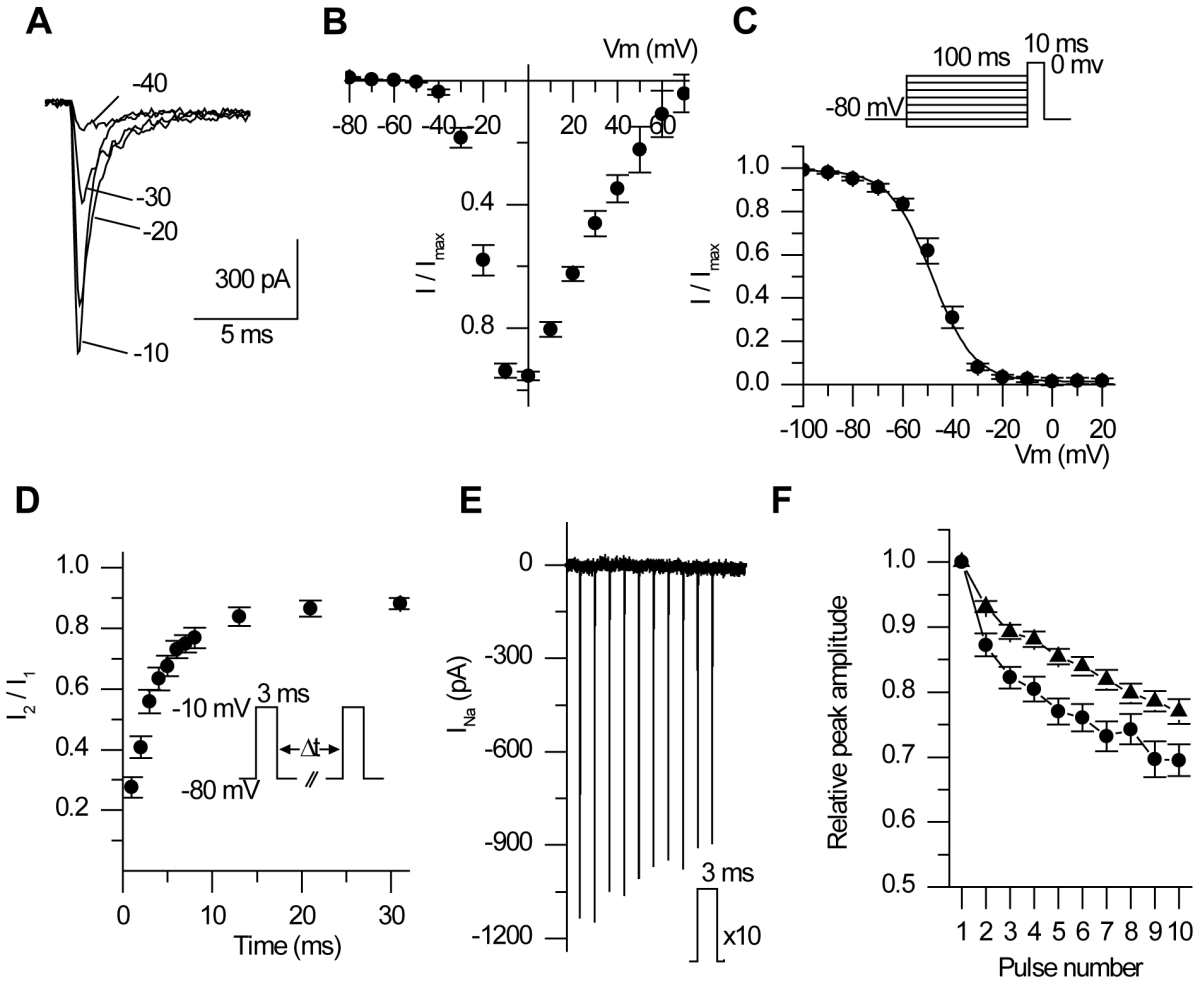
Properties of the voltage-gated sodium current from honeybee ALNs. A, Sodium current traces obtained from an ALN for voltage steps to the indicated potential from a holding potential of −80 mV. B, Mean relative I/V curve (n = 24). C, Mean steady-state inactivation curve (n = 12). D, Recovery from fast inactivation in ALN (n = 12). E, Use-dependent decrease in the amplitude of the sodium current observed in an ALNs submitted to a 10-pulses train at 13 Hz. I, Average decrease in the amplitude of the sodium current in ALNs submitted to a 10-pulse train at 13 Hz (filled triangles, n = 36) or 35 Hz (filled circles n = 7).

In ALNs, the mean current-voltage curve peaks at 0 mV (Figures 1A and B) and at this potential, the mean maximal current amplitude is −121±12 pA/pF (n = 24). Individual I/V curves were fitted (Eqn 1) and on average, the potential for half activation (V_m_) and the slope factor (K_m_) are −18.9±1.0 mV and 5.2±0.2 respectively (n = 24). The mean parameters of the steady-state inactivation curve (Figure 1C) were obtained by fitting a Boltzmann equation to experimental data from individual ALNs (Eqn 2). On average, the potential for half inactivation (V_h_) and the slope factor (K_h_) are −46.9±1.7 mV and 7.2±0.3 respectively (n = 12). The time course of the sodium current was characterized by measuring the time to peak (t_p_) and the time of half inactivation (t_h_). On average, t_p_ decreases from 0.81±0.03 ms at −20 mV to 0.46±0.02 ms at +30 mV (n = 24). On average t_h_ decreases from 0.77±0.08 ms at −20 mV to 0.17±0.01 ms at +30 mV (n = 24). The recovery from inactivation was explored using a two-pulse protocol (Figure 1D) and data from individual ALNs were fitted with a two exponential function. A fast time constant (τ_1_ = 2.6±0.2 ms, n = 12) accounts for 75% of recovery and a slow time constant (τ_2_ = 69±18 ms, n = 12) is responsible for the rest of the recovery. We next assessed the effect of repeated short depolarizations (ten 3ms-pulses, Figure 1E), mimicking those encountered by neurons during trains of action potentials. On average (Figure 1F), the sodium current amplitude is significantly reduced to 77±2% of its control value after ten pulses at 13 Hz (n = 36). Under a higher stimulation frequency i.e. 35 Hz, the peak is significantly more reduced and reaches 70±2% of its control value (n = 7).

### Effects of pyrethroids on ALNs

**Figure 2:**
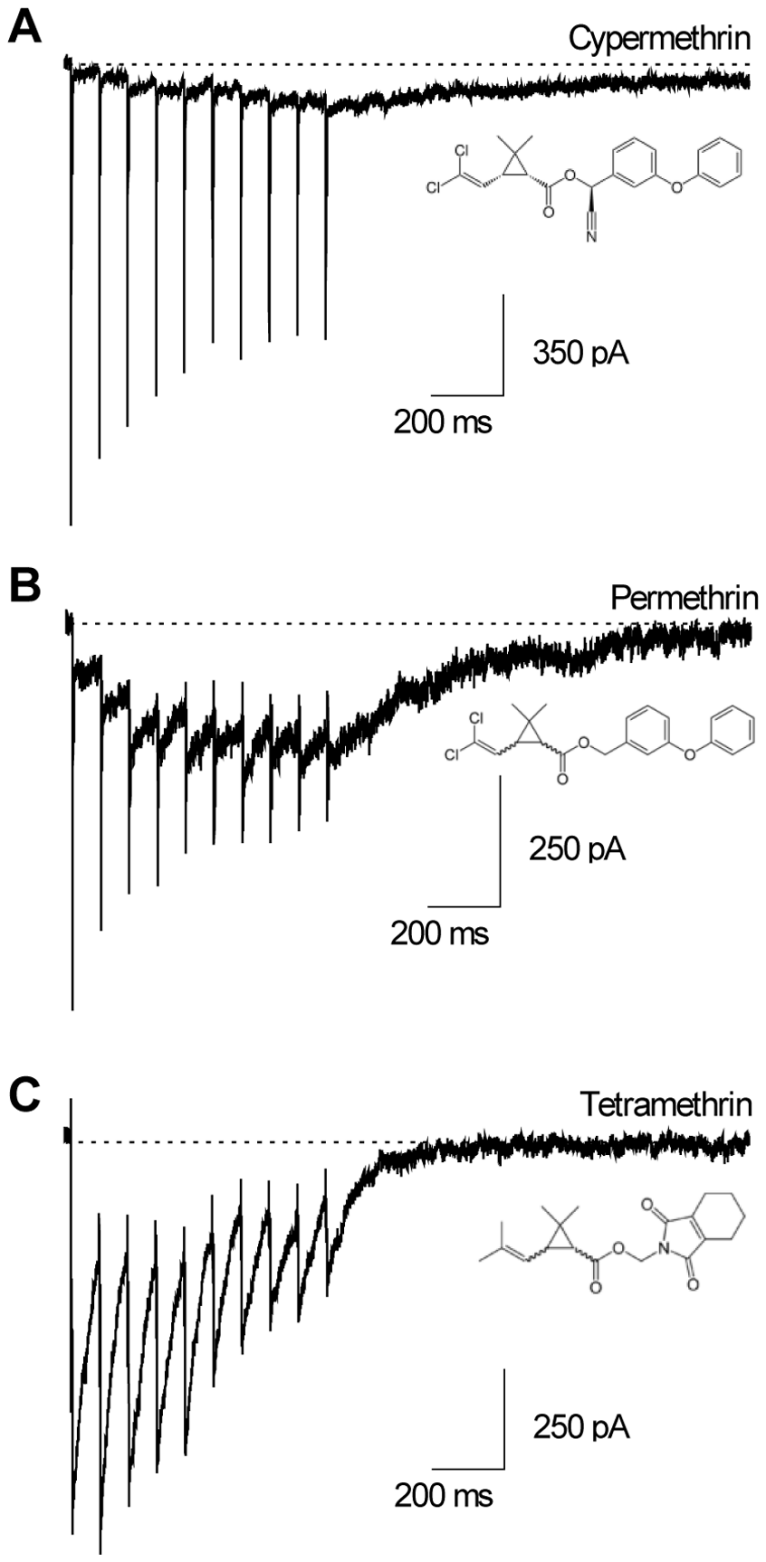
Use-dependent effects of type II (cypermethrin) and type I (permethrin and tetramethrin) pyrethroids in ALNs. Sodium current recordings in response to a 10-pulse train (3 ms, from −80 mV to −10 mV, 13 Hz) in three different ALNs in the presence of 10 µM cypermethrin (A), 10 µM permethrin (B) or 10 µM tetramethrin (C).

**Figure 3:**
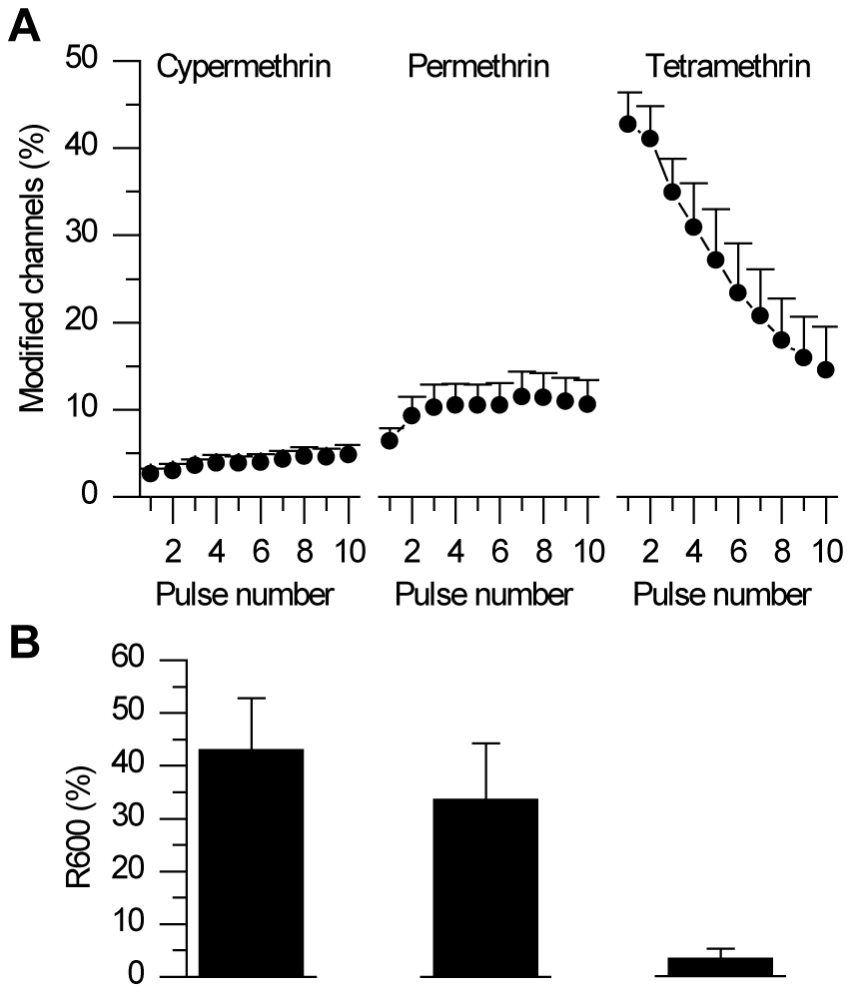
Use-dependent sommation of modified channels and tail current inactivation kinetics in ALNs. Mean time course of pyrethroid-modified channels along with protocol shown in Figure 2 (n = 7, 10 and 10 ALNs for cypermethrin, permethrin and tetramethrin, respectively). The percentage of modified channels is calculated from tail currents amplitude according to Eqn 3. Whereas cypermethrin and permethrin induce an increase in % of modified channels, tetramethrin shows the opposite effect. B2. Kinectics of tail currents estimated by the R600 value, i.e. the percentage of residual tail current 600 ms after the end of the tenth pulse of the 10-pulse protocol. Tetramethrin induces faster decaying tail currents than cypermethrin or permethrin.

The effects of pyrethroids were quantified using repetitive depolarizations (3 ms, 13 Hz) to mimic trains of action potentials encountered during neuronal activity. Figure 2 shows sodium currents recorded in the presence of 10 µM of cypermethrin, permethrin or tetramethrin. The membrane potential was stepped to −10 mV from a holding potential of −80 mV and the effects of pyrethroids were assessed 3 minutes after the beginning of exposure. Peak current as well as tail current amplitudes were measured. All pyrethroids induced the appearance of a prominent tail current upon repolarization from each step of the train (compare with control currents in Figure 1E), revealing a slowing-down of channels deactivation (Figure 2). Cypermethrin elicits a progressive tail current summation in all neurons (n = 7). With permethrin, the majority of neurons (7 out of 10) showed also a progressive summation of the tail current, at least for the first pulses. However, a stationary value (2 neurons out of 10) as well as a decreasing value (1 neuron out of 10) could also be recorded along with successive steps. Interestingly, in the presence of tetramethrin, the tail current amplitude already reached its maximum after the first or second pulse and amplitude then decayed to a smaller value in all neurons (n = 9). In single neurons, the fraction of channels modified by pyrethroids out of the total active channel population is traditionally calculated from tail current amplitudes using Eqn 3. The percentage of modified channels significantly increases from 3±1 to 5±1% (n = 7, p<0.05) with cypermethrin (Figure 3A). With permethrin, the percentage of modified channels is 6±1% after the first pulse and 11±3% after the tenth pulse (n = 10 neurons) but these values are not significantly different. With tetramethrin, the average percentage of modified channels decreases by a factor of three between the first and the tenth pulse (the mean value drops from 43±4 to 15±5% n = 9, p<0.001). Therefore while significantly different percentages of modified channels are obtained at the first pulse for the three compounds (tetramethrin> permethrin> cypermethrin), this difference tended to attenuate with activity. We then estimated the decay-rate of the tail currents induced by each compound by measuring the remaining tail current amplitude 600 ms after the end of the tenth pulse (R600). After that delay, the residual tail current is decreased to 43±10% of its initial value for cypermethrin (n = 7, p<0.05, Figure 3B), 34±11% for permethrin (n = 10, p<0.05) and 3±2% of its initial value for tetramethrin (n = 10, p<0.05), Tetramethrin thus produced significantly faster decaying tail currents than permethrin and cypermethrin (p<0.05 and p<0.01 respectively).

**Figure 4:**
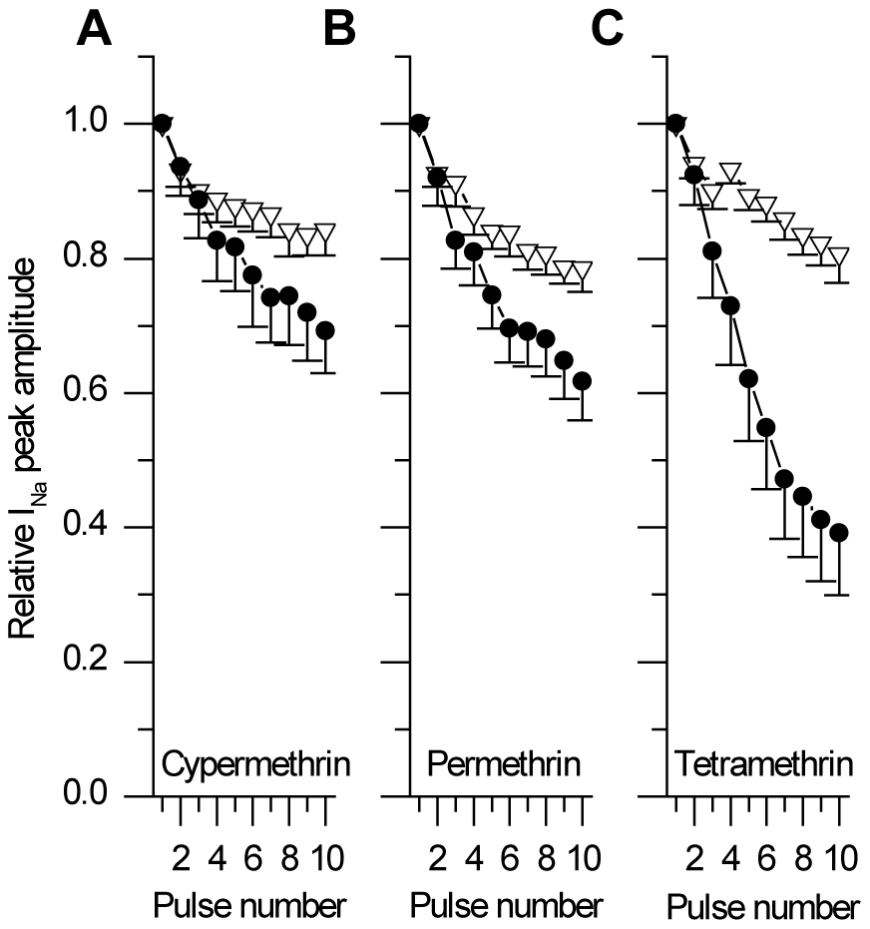
Effect of pyrethroids on the use-dependent decrease in the sodium current peak. Mean evolution of the relative amplitude of the peak current along with protocol shown in Figure 2 (current normalized to the amplitude obtained at the first pulse of the train) from ALNs in a control solution (empty triangles) and after exposure to pyrethroids (filled circles). All pyrethroids tested amplify the decrease in current amplitude and tetramethrin (n = 10) has a stronger effect than cypermethrin (n = 6) or permethrin (n = 10).

Not only a prominent effect of pyrethroids was observed on tail currents, but use-dependent effects were also observed on the peak of the sodium current during depolarization. While incubation with cypermethrin and tetramethrin significantly reduce the peak sodium current amplitude obtained in response to the first depolarization by 33±4% (p<0.05, n = 13), and 6±11% (p<0.05, n = 10), respectively, this effect was not significant with permethrin. Moreover, all pyrethroids accelerated the cumulative inactivation of the sodium current peak seen in control conditions (Figure 1E and F). As shown in Figure 4, the peak current decreases to 62, 69 and 77% of its initial values, levels that are significantly different from the level reached in control (p<0.01 for permethrin and tetramethrin, p<0.05 for cypermethrin). The use-dependent sodium current decrease is thus significantly more important with tetramethrin than with permethrin or cypermethrin (p<0.05).

**Figure 5:**
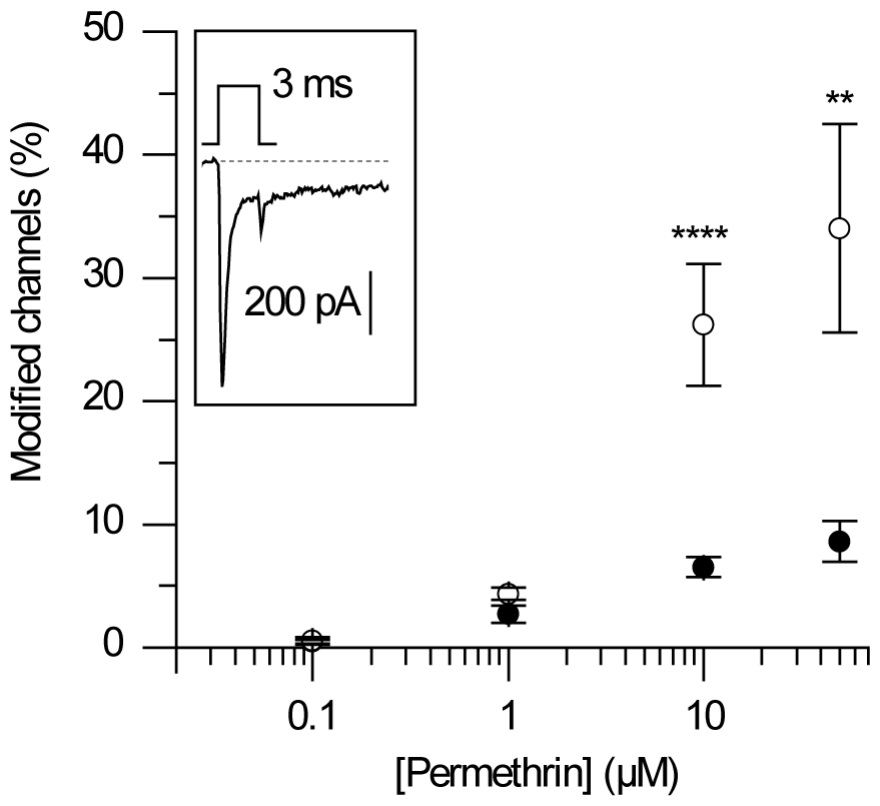
Dose-response curve of permethrin-modified sodium channels in ALNs. Percentage of permethrin-modified channels, calculated according to Eqn 3, from the permethrin-induced tail current amplitude measured 3ms after a single pulse (of 3 ms in duration, see inset). The percentage of modified channels increases as a function of permethrin concentration (filled circle, n = 7, 10, 25 and 9 at 0.1, 1, 10 and 50 µM respectively). The same curve but recorded in ORNs (from Kadala et al. 2011) is shown for comparison (empty circles, n = 3, 9, 14, 5).

In addition to use-dependent modifications, we explored dose-dependent effects of permethrin in ALNs and compared the results to our former study made in peripheral neurons (ORNs). A dose-dependent increase in modified channels was obtained in ALNs as well as in ORNs (Figure 5) in response to a single depolarization. As compared to ORNs, lower levels of modification were obtained in ALNs for concentrations of 10 and 50 µM, suggesting a stronger sensitivity of peripheral *vs* central channels to permethrin [12].

### Pyrethroids affect transitions to channel open and inactivated states

**Figure 6:**
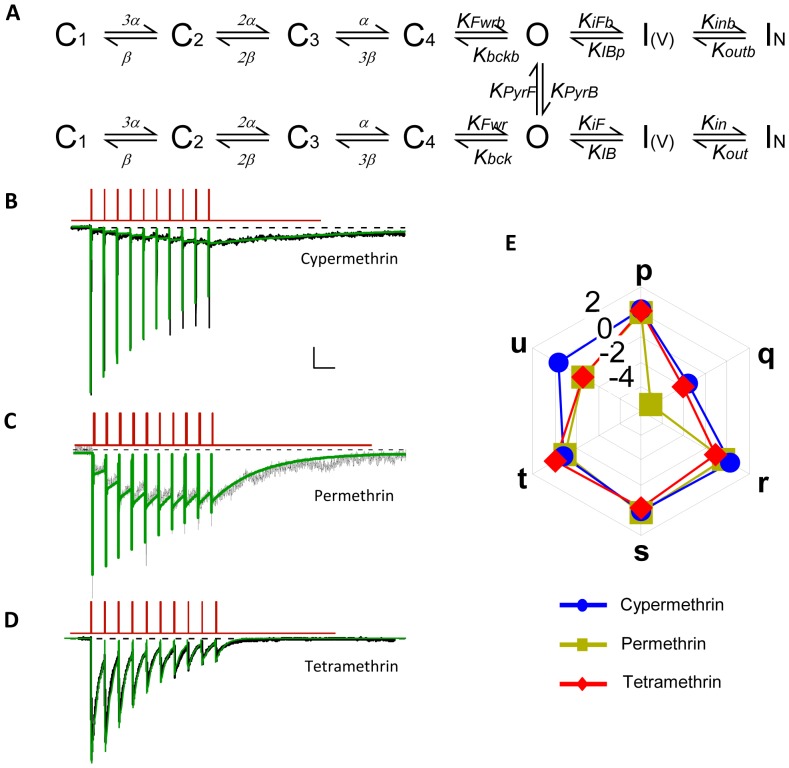
Pyrethroids decrease deactivation rate constants. A. State model used to fit the experimental traces (see Methods and text for details). Pyrethroids can only bind to open channel. Once bound they modify channel kinetics to and from open and inactivated states by given factors, i.e. KFwr, Kbck, KiF, KIB, Kin and Kout by p, q, r, s, t, and u respectively to give KFwrb, Kbckb, KiFb, KIBb, Kinb and Koutb. Voltage dependence is not changed. B-C-D. Fitting of experimental traces obtained under differents pyrethroids (cypermethrin, permethrin, tetramethrin) using the above model allows to determine the changes in the different kinetic parameters. Scale bar 200 pA (B and C) or 500 pA (D) and 150 ms. E. Radargraph of the changes (logarythmic scale) in the different kinetic parameters produced by pyrethroids. Fitting for KpyrF KpyrB, the binding and unbinding rate constants at a single concentration (10 µM), are used to calculate Kd.

A markovian 7 states channel model was set-up to analyze more precisely the effect of pyrethroids (Figure 6A, see Materials and Methods). Four Close states were linked by identical voltage-dependent transition rates (forward α and backward β). Forward and backward transition rates to Open and Fast inactivation were also voltage-dependent, while those to slow–pore-dependent-inactivation were set as constant (see methods). Fit of this model to experimental data recorded in control conditions gave a set of values that allowed to mimic kinetics of sodium current traces as well as activation and inactivation curves and behavior of the channel during a train of depolarizations (Figure S1). The model allowed the binding of the drugs to the Open state to promote a modified channel behavior where the rate constants to and from the Open and Inactivated states are modified by a specific factors (p, q, r, s, t, u respectively), while purely voltage-dependent transitions between closed states remained unaffected (Figure 6A). This model was selected because it fits with previous observations of pyrethroids effects on sodium channels and because the 2 pyrethroids binding-sites resides in the pore-domains with interactions with segments S5 and S6 of domains I, II and III (II-S5 and III-S5-S6) and with the loop connecting segments 4 and 5 in domains I and II (I-L45, II-L45) close to the activation and inactivation machinery but far apart from the voltage sensor S4 [28], [29]. Although binding to closed state could theoretically occur [30], we found that it was not necessary in our experimental conditions (see Methods, Discussion and Figure S3). We then use the data from Figure 2 in the presence of cypermethrin, permethrin or tetramethrin to evaluate the respective changes produced by the 3 drugs. Figures 6B, C, D show, super-imposed, the experimental data, in black and the fitted currents using this model in green. Forward transition to open, inactivated and deep-inactivated states were barely affected (p, r, s, Figure 6E). As expected from the effects on deactivation, the most affected rate constant was the closing rate constant from Open to Closed state C4, Kbck, which was decreased by several orders of magnitudes (q values). In the case of permethrin this rate was almost completely nullified. Together with a severe reduction in the backward transition from deep inactivation (factor u), this explained the slow tail current recorded, and the cumulative inactivation that can be seen for longer train of depolarization (Figure S2). The transition rate back from slow-inactivation (factor u), was also strongly decreased for tetramethrin, thus increasing cumulative inactivation, favoring the decrease of the currents during stimulation and preventing the slow and moderate development of the cumulative tail current seen with cypermethrin (Figure 6B). Finally, drug binding and unbinding (kPPyrF and KpyrB) to the open state were increased in the case of tetramethrin when compared to cypermethrin or permethrin (by 1 and 2 order of magnitude). In all cases however estimated Kd values were of similar order of magnitude (as seen experimentally [32]).

## Discussion

In this study, we have for the first time successfully (i) characterized the use-dependent effects of pyrethroids on central olfactory neurons and (ii) mathematically modeled currents modifications. Whereas the action of pyrethroid insecticides on voltage-gated sodium channels has already been investigated in invertebrates [22], [23], [24], [33], [34], [35], data on beneficial insects such as bees are lacking, while precise molecular actions of these insecticides are needed to interpret and predict their sublethal effects, as well as those of insecticides with a similar mode of action. Our examination of the effects of pyrethroids on cultured neurons from a key area involved in olfactory coding and processing (antennal lobes) yielded several major informations that are key to understand their mechanisms and specificity.

In ALNs, the first observation made with all 3 pyrethroids is a marked slowing of the tail current. In addition we also reported drug-induced use-dependent modifications of current peak and current tail, that have previously been proposed to result from (i) accumulation of persistently open channels [17] (ii) the progressive recruitment of channels after they cycle from open to inactivated state [24], [36], [37], or (iii) recruitment of silent channels by type I pyrethroids that could also be pictured as a progressive enrollment of formerly silent pyrethroid-hampered sodium channels [12]. These use-dependent decrease in the peak current, and summation of the tail currents are strongly dependent on the chemical nature of the drug. In addition, the peak of the current elicited by a single pulse was decreased by a 3 minutes exposure to cypermethrin or tetramethrin but not permethrin. This behavior was specific of central neurons (this study and [14]) since tetramethrin and permethrin do not decrease the current peak in ORNs from antennae (at least at the time scale of our experiments), whereas they increase the late part of the current, nevertheless suggesting a slowing-down of the current inactivation and/or activation kinetics during the depolarizing voltage step [12].

Our kinetic analysis of these effects allows to better understand the transitions rates that are affected by the different pyrethroids, and thus help us to understand their specificity. First, it is very interesting to notice that the effects of all 3 drugs can be appreciated using the same mathematical model allowing binding of the drug to the Open state only. The fact that tetramethrin can affect channel behavior at the first pulse is correlated in our case to the difference in the drug binding and unbinding rate constants (2 orders of magnitude larger for tetramethrin than for cypermethrin, not shown) and does not require any binding to closed channels as intuitively expected and experimentally tested (Figure S3). These differences in the rate constants may be related to any drug specific differences in solubility, steric hindrance or differential binding to the 2 sites identified in the channel structure [28], [29]. However, more experimental and modeling data are clearly needed to unequivocally rule-out binding to closed channel. Second, it is clear that all 3 drugs slowed the tail currents and markedly decreased the deactivation rate. In ALNs, the type II pyrethroid cypermethrin produces a slower tail current than the type I pyrethroid tetramethrin, while permethrin, another type I pyrethroid, produced an intermediary behavior with faster tail currents as already seen in antennal ORNs [12], but partially cumulative, like cypermethrin. The kinetic analysis of these currents demonstrated that (i) all 3 pyrethroids decreased deactivation rate Kbck by several order of magnitudes (factor q in our model) and (ii) that type I pyrethroids only can decrease specifically the transition rate from deep-inactivation, Kout (factor u), preventing any long-term cumulative tail current as those seen with cypermethrin. Tetramethrin in addition also increased, although moderately, the rate constant driving to slow pore-inactivation (factor t) when compared with permethrin, thus producing a stronger cumulative inactivation and the marked decrease in the tail currents recorded during the trains of depolarizations. These differences are better evidenced when simulation are performed with higher pyrethroids concentrations or longer trains of depolarizations (see Figure S2), and thus provide informations for further experimental tests. They also suggest that channels may accumulate in a slow inactivating state in a drug- and rate-dependent fashion thus challenging the notion of modified channels as usually calculated from the ratio of the tail over the peak conductances.

These differential effects of the three pyrethroids on use-dependent parameters may be related to the existence of multiple binding sites within the channel pore as suggested earlier [29]. However, the fact that the calculated Kd are quite similar between the 3 drugs and that the same kinetic parameters seem to be affected (in the limit of our modeling), suggest also that the binding site(s) may be (all) located in a strategic place where activation, fast- and slow-inactivation can be affected more or less directly. Clearly the 2 identified binding-sites comprising IL45-IS5-IIS6 and IIL45-IIS5-IIIS6 [29], [38] fit with these requirements. The phenyl group of cypermethrin or permethrin can be docked between the linker and the S5 and the S6 transmembrane helices constituting the major binding site(s), and placing the di-methylcyclopropane and the CCl moeties of permethrin or cypermethrin groups at specific position below or above the gating hinge, close to the pore helix. These specific binding may ensure the drug-dependent modifications of channel properties including the effects on deactivation, inactivation (action on the gating hinge) or pore-inactivation (action on the pore helix). In this case the presence of a cyano group in cypermethrin would impede any effect on pore-inactivation, since t and u factors are poorly affected (∼1). Functional analysis of wild type and mutated channels in expression system with different pyrethroids and using a modeling approach similar to this one may therefore bring important information about the structure and mechanisms underlying this modulatory process.

In bees, the differences in tail current decay rates that we observed between type I and type II pyrethroids are reminiscent of dissimilarities in nerve electrical activity after exposure to these compounds: repetitive discharges for type I pyrethroids [39] and prolonged depolarization for type II pyrethroids [40]. However it should be noted that, although symptoms of poisoning by type I and type II pyrethroids may look different, they both eventually lead to paralysis or prostration in exposed animals [6]. Central and peripheral honeybee neurons also behave in a different manner with regard to cumulative inactivation. Whereas pyrethroids enhance the activity-related decrease of the peak current in ALNs, the opposite effect was suggested earlier in ORNs [12] thus producing a progressive reduction in the ALNs activity but a sustained depolarization in ORNs. Pyrethroids may then differentially impair the firing pattern in the two compartments of the honeybee olfactory pathway. Interestingly, a differential sensitivity to deltamethrin was found in splice variants from German cockroach *Blatella germanica* Na^+^ channel gene [41]. If honeybee, like other insects has only a single gene coding for voltage-gated sodium channels (*para*), the molecular basis for this specific behavior may result either from the existence of alternative splicing and/or RNA editing of this gene [42], [43] and/or from the co-expression of different sets of accessory subunits, since 5 TipE genes homologs have been identified in insects [44]. All these combinations of sodium channel subunits can potentially display specific gating properties, modulation and pharmacological sensitivities. Analysis of the tissue-specific expression of variants of the pore subunit and/or regulatory subunits of voltage-gated sodium channels remains to be explored in *A. mellifera* but will certainly bring key information to understand the differential effects recorded in ALNs and ORNs. Entry of sodium channels into slow inactivation occurs both by long conditioning depolarizing steps or by repeated short pulses [45], [46]. The fact that pyrethroids also affected slow inactivation in ALNs strongly support their role in modulating action potential firing during neuronal activity [46]. Our data underline the broad functional action spectrum of pyrethroids in bees, re-emphasizing the complexity of the differential biophysical modifications they can induce. Not only pyrethroids can induce functionally different modifications in separate populations of sodium channels, but their separate mode of action on other targets, especially calcium channels [47], [48], [49], reinforce their potency to induce sublethal effects on both pests and bees under exposure to weak doses.

Our study further documents the mechanisms by which pyrethroids lead to sublethal neural effects in the honeybee. A variety of subtle symptoms might be triggered by differential effects in peripheral and central neurons and compound-specific abilities to alter sodium channels. For instance, under sublethal exposure, learning performances were differentially affected by pyrethroids and odor training responses were the most affected [8], [9]. In summary, in central and peripheral bee neurons, pyrethroids yield strong use-dependent effects of different nature. The pharmacological effects of pyrethroids on activity-related adaptation in peripheral ORNs and central antennal lobe neurons involve separate processes which can potentially impair detection and information processing in the olfactory pathway of the bees.

## Supporting Information

Figure S1 Fitting experimental data with the channel-state-model in control conditions allows to evaluate all parameters in control conditions. With these parameters, current traces (A, B), current–voltage curve (C, V_act_ = −22 mV, k = 9.2 mV), as well as progressive use-dependent current decrease during train of depolarizations (D) are correctly simulated (green traces channel model, black traces experimental data).
(TIF)

Figure S2 Numerical simulation of the model channel behavior. Simulation was performed during longer trains of depolarizations (30 pulses), in standard pyrethroid concentration (10 µM, green) and for 10 fold lower (red) or higher (blue) pyrethroid concentrations underlines the differences between the 3 drugs that can be further tested experimentally.
(TIF)

Figure S3 Fitting experimental data with the state-model incorporating binding to Open and/or Closed states. Modification in the rate constants using the state model on top and allowing binding of tetramethrin to Open or Closed states only or to both states. In the 3 cases the changes in rate constants were qualitatively similar, only Kds value were different, with respective Kd of 65 nM and 6.1 µM for binding to Open or Closed channels exclusively, or 61 nM and 15 µM for possible binding to Open and Closed states simultaneously.
(TIF)
