# Supplementary figures and images for: Pyrethroids Differentially Alter Voltage-Gated Sodium Channels from the Honeybee Central Olfactory Neurons
Source: PLoS One. 2014 Nov 12;9(11):e112194. doi: 10.1371/journal.pone.0112194 (PMC4229128; doi:10.1371/journal.pone.0112194)

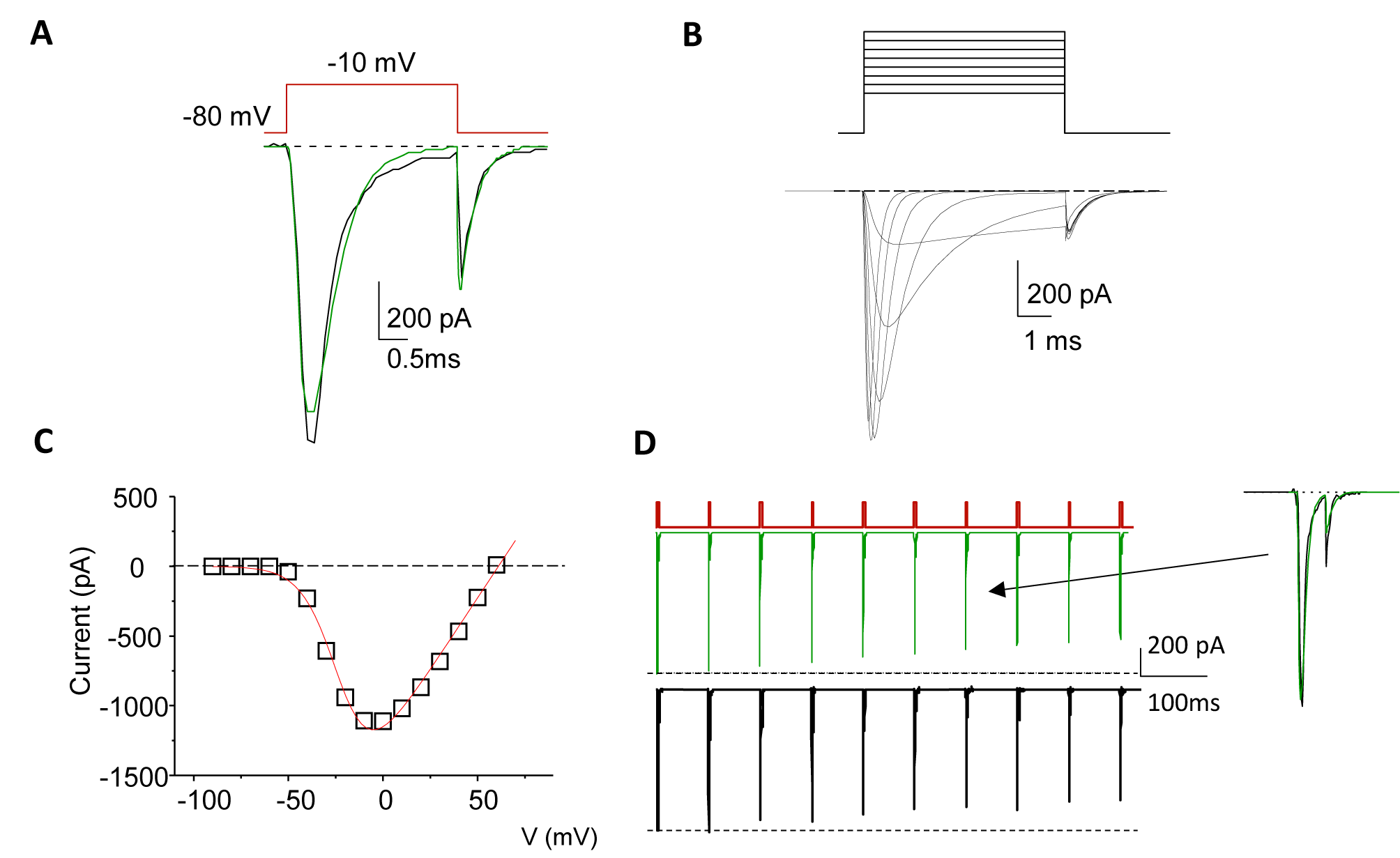

Supplement: Figure S1 — Fitting experimental data with the channel-state-model in control conditions allows to evaluate all parameters in control conditions. With these parameters, current traces (A, B), current–voltage curve (C, Vact = −22 mV, k = 9.2 mV), as well as progressive use-dependent current decrease during train of depolarizations (D) are correctly simulated (green traces channel model, black traces experimental data). (TIF) [file pone.0112194.s001.tif]
